# Supplementary material for: Regulation of sleep plasticity by a thermo-sensitive circuit in Drosophila
Source: Sci Rep. 2017 Jan 13;7:40304. doi: 10.1038/srep40304 (PMC5233985; doi:10.1038/srep40304)
Supplement: Supplemental Information [file srep40304-s1.pdf]

Regulation of sleep plasticity by a thermo-sensitive circuit in *Drosophila*

Angelique Lamaze[1], Arzu Öztürk-Çolak[2], Robin Fischer[3], Nicolai Peschel[3], Kyunghee Koh[2] and James E.C. Jepson[1*]

[1] UCL Institute of Neurology, London, UK

[2] Department of Neuroscience, the Farber Institute for Neurosciences, and Kimmel Cancer Center, Thomas Jefferson University, Philadelphia, USA

[3] Neurobiology and Genetics, Biocenter, University of Würzburg, Würzburg, Germany

SUPPLEMENTAL INFORMATION

FIGURE S1

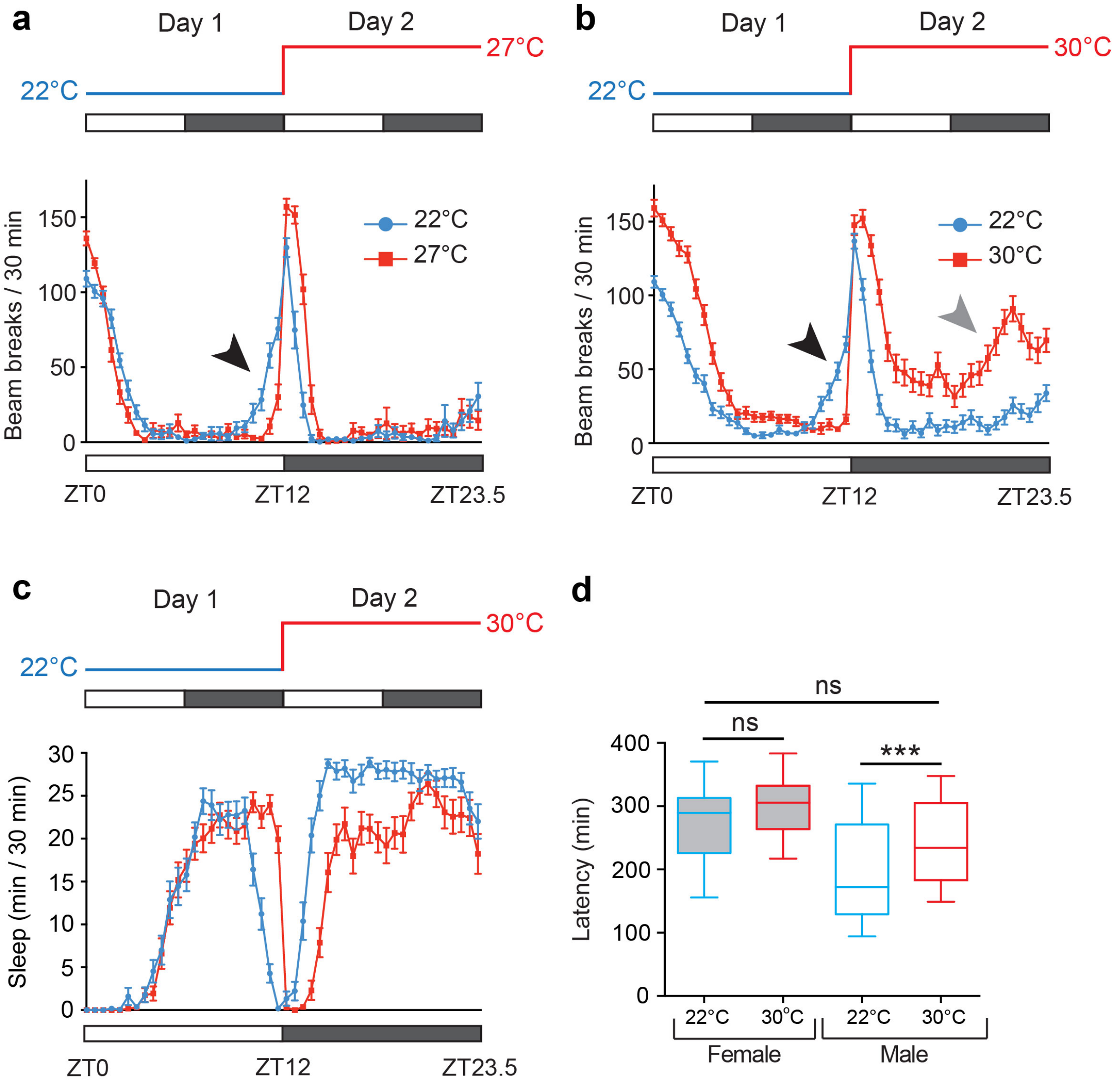

Fig. S1: Alterations in locomotion and sleep architecture due to increased ambient temperature in *Drosophila* males and females. (**a**) Average number of beam crossings per 30 min over 24 h for wild-type males shifted from 22°C to either 27°C (**a**) or 30°C (**b**). Black arrowheads indicate a delay in evening anticipation observed at either 27°C or 30°C relative to 22°C. Grey arrowhead denotes advanced morning anticipation observed at 30°C, but not at 27°C. (**c**) Average sleep patterns of adult mated female flies shifted from 22°C to 30°C. n =

32. (**d**) Latency to initiate the first day sleep bout in adult males and females shifted from 22°C to 30°C. ***$p < 0.0005$, ns – $p > 0.05$, Kruskal-Wallis test with Dunn's post-hoc test.

FIGURE S2

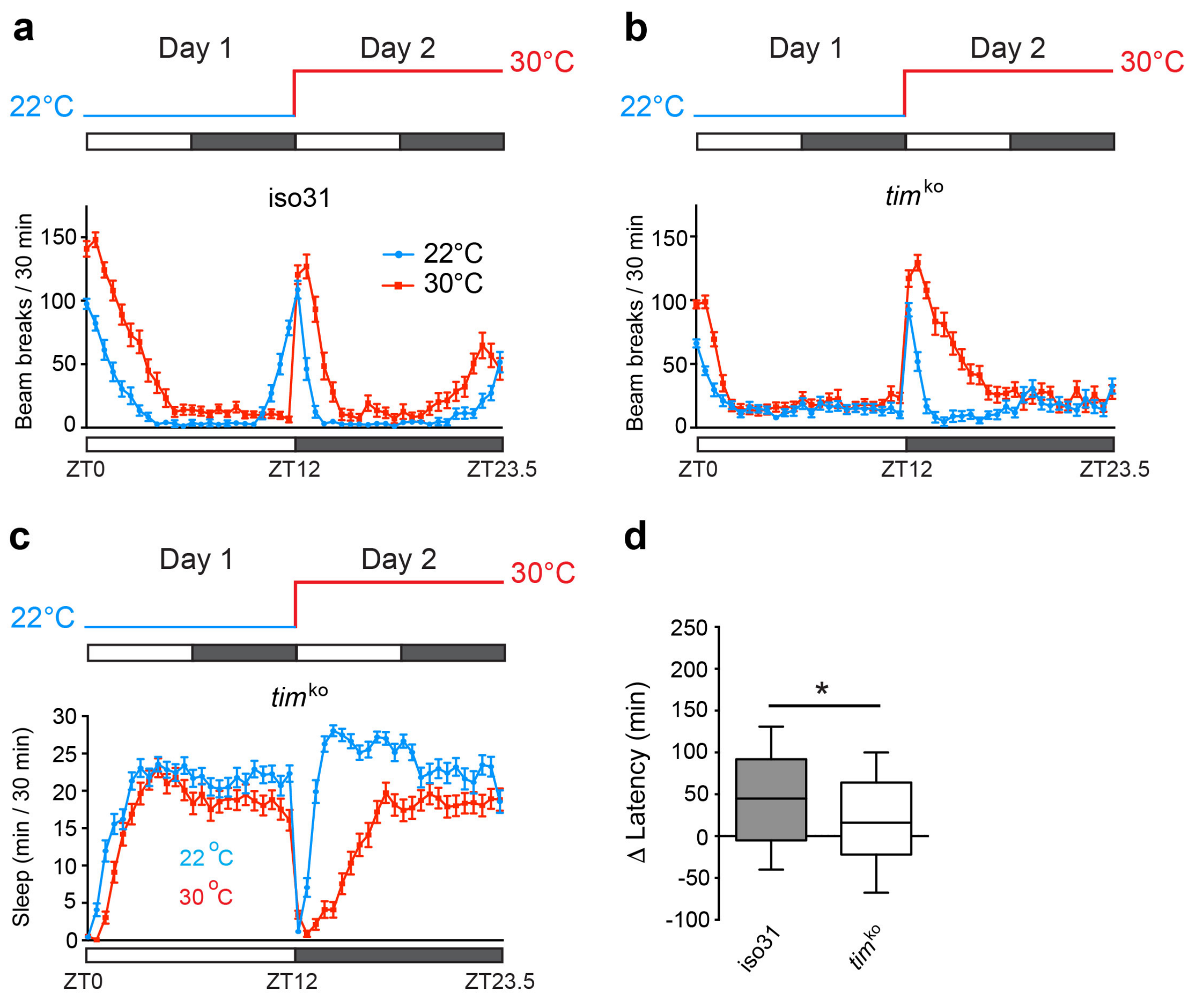

Fig. S2: Locomotor and sleep patterns of *timeless* null flies during warm conditions. (a-b) Number of beam breaks per 30 min in wild type iso31 controls (n = 31) or *timeless* null (*tim*^{ko}) homozygotes (n = 33) during consecutive days at either 22°C or 30°C. Locomotor activity was measured under 12 h light: 12 h dark conditions (white/grey bars) with Zeitgeber Times (ZT) shown below. Data are presented as mean ± SEM for each time point. (**c**) Sleep patterns of *tim*^{ko} homozygotes at either 22°C or 30°C. (**d**) Change in latency to the first day sleep episode in iso31 controls (n = 69) or *timeless* null (*tim*^{ko}) homozygotes (n = 64) following a shift from 22°C to 30°C. *$p < 0.05$, Mann-Whitney U-test.

Figure S3

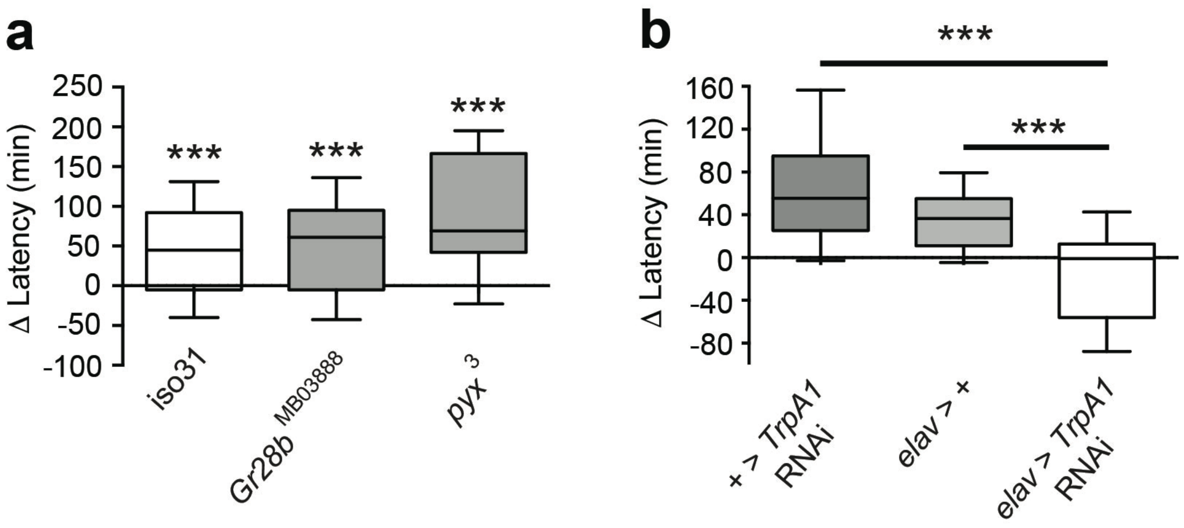

Fig. S3: The Gr28b and Pyrexia thermo-receptors do not mediate PMW. (**a**) Change in latency to the first day sleep episode in iso31 controls (n = 69), *Gr28b*^{MB03888} (n = 27) or *pyrexia*^{3} (*pyx*^{3}; n = 26) homozygotes following a shift from 22°C to 30°C. All genotypes show a significant increase in latency following an increase in ambient temperature to 30°C. ***p < 0.0005, compared to a median of zero, Wilcoxon signed rank test. (**b**) Pan-neuronal knockdown of TrpA1 expressing using a *UAS-TrpA1* RNAi transgene under control of *elav*-GAL4 suppresses PMW. n = 33-120. ***p < 0.0005, Kruskal-Wallis test with Dunn's post-hoc test.

FIGURE S4

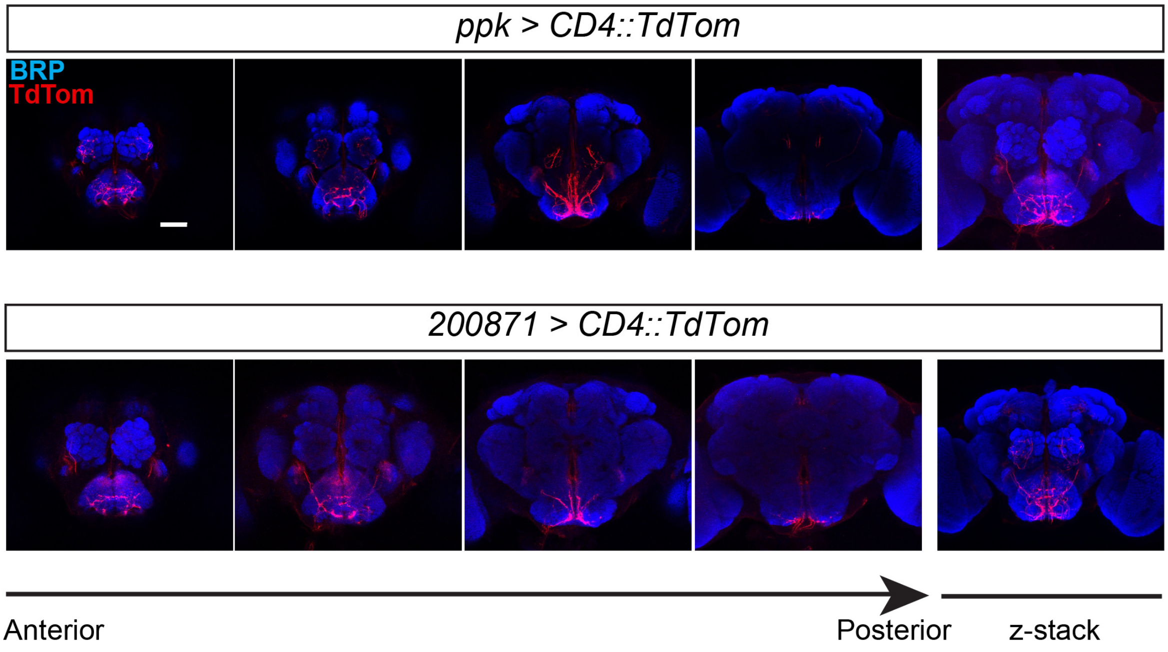

a
ppk > CD4::TdTom
BRP
TdTom
200871 > CD4::TdTom
Anterior
Posterior
z-stack

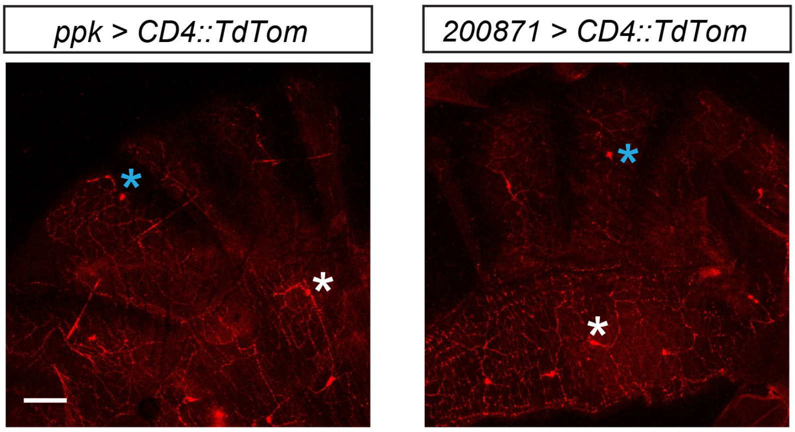

b
ppk > CD4::TdTom
200871 > CD4::TdTom
*
*
*
*

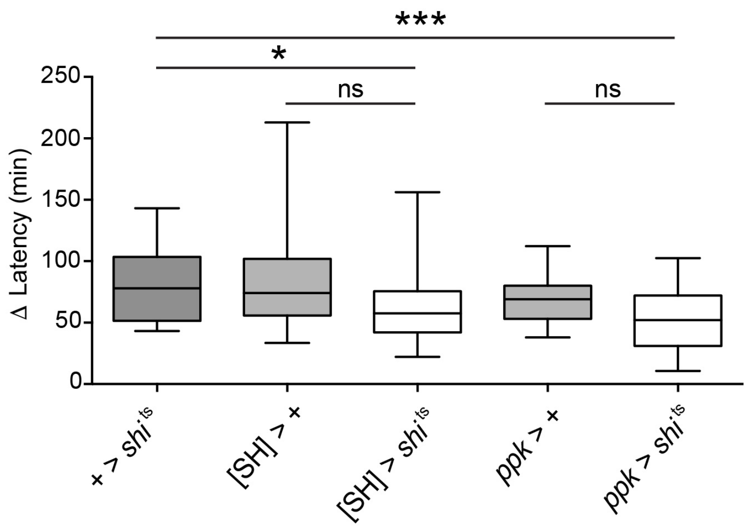

c
***
*
ns
ns
Δ Latency (min)
250
200
150
100
50
0
+ > shi^ts
[SH] > +
[SH] > shi^ts
ppk > +
ppk > shi^ts

Fig. S4: ***pickpocket*****-expressing class IV multi-dendritic (mdIV) neurons do not mediate PMW.** (**a**) Confocal slices showing expression patterns of *ppk*- and *ppk*[200871]-GAL4 along the anterior/posterior axis of the adult brain. Right, z-stack projections. Neurons are labeled using a UAS-CD4::TdTom transgene (Red). Synaptic neuropil is labeled using an anti-Bruchpilot (BRP) antibody. Scale bar: 50 μm. (**b**) Visualization of mdIV neurons on the adult body wall. White stars: v'ada neurons; blue stars: ddaC neurons. Scale bar: 100 μm. (**c**) Acute inhibition of synaptic output (using UAS-shi^{ts}) from *TrpA1*[SH]- and *ppk*-neurons does not suppress the delay in initiation of night sleep in response to increased ambient temperature. n = 24-77. $^{*}p < 0.05$, $^{***}p < 0.0005$, ns – $p > 0.05$, Kruskal-Wallis test with Dunn's post-hoc test.

FIGURE S5

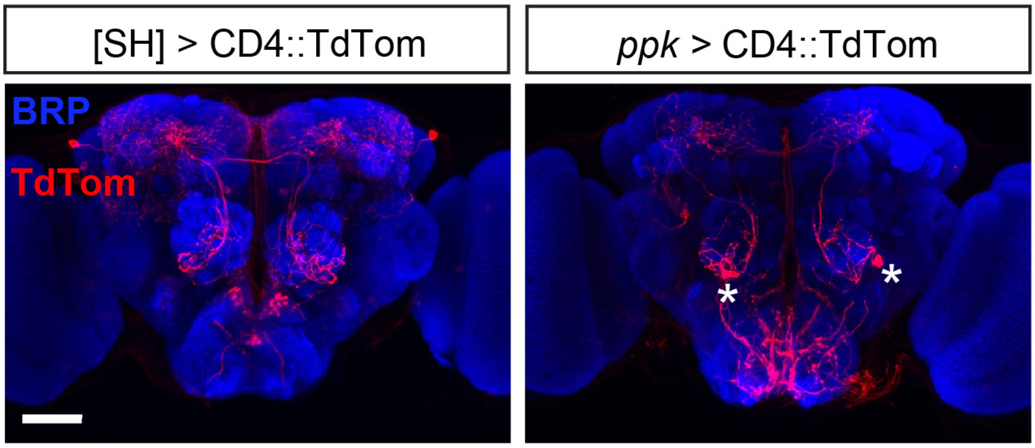

Fig. S5: The *ppk*-GAL4 driver stochastically labels AC neurons in the adult *Drosophila* brain. Confocal z-stacks showing *TrpA1*[SH]- and *ppk*-neurons labeled using UAS-CD4::TdTom (TdTom). Synaptic neuropil is labeled using an anti-Bruchpilot (BRP) antibody. Scale bar: 50 µm. Star: AC neuron cell bodies observed when labeling *ppk*-neurons.

FIGURE S6

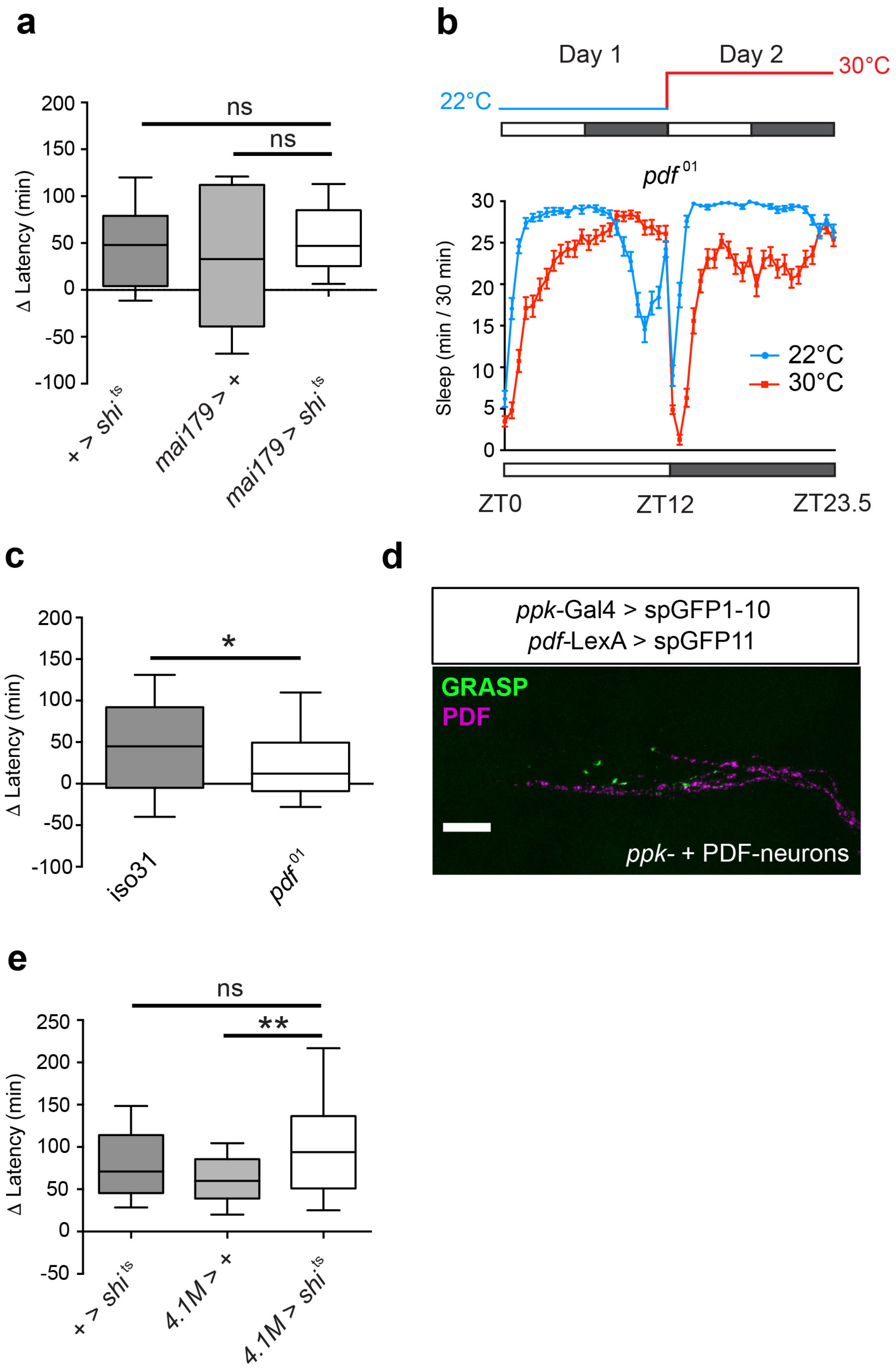
a
Δ Latency (min)
200
150
100
50
0
-50
-100
ns
ns
+ > shi ts
mai179 > +
mai179 > shi ts
b
Day 1
Day 2
22°C
30°C
pdf 01
Sleep (min / 30 min)
30
25
20
15
10
5
0
22°C
30°C
ZT0
ZT12
ZT23.5
c
Δ Latency (min)
*
iso31
pdf 01
d
ppk-Gal4 > spGFP1-10
pdf-LexA > spGFP11
GRASP
PDF
ppk- + PDF-neurons
e
Δ Latency (min)
250
200
150
100
50
0
-50
ns
**
+ > shi ts
4.1M > +
4.1M > shi ts

Fig. S6: CRY-positive LN_d and PDF neurons are not necessary for PMW. (**a**) Acute inhibition of synaptic output (using UAS-*shi*^{ts}) from CRY-positive LN_d neurons does not suppress the delay in initiation of night sleep in response to increased ambient temperature. + > UAS-*shi*^{ts}: n = 25; *mai179* > +: n = 7, *mai179* > *shi*^{ts}: n = 21. (**b**) Average sleep patterns of adult *pdf*^{01} homozygote males shifted from 22°C to 30°C at ZT0. (**c**) Comparison of PMW between iso31 controls and *pdf*^{01} homozygotes. iso31: n = 79, *pdf*^{01}: n = 65. Iso31: p < 0.0001; *pdf*^{01}: p = 0.0007, Wilcoxon signed rank test. (**d**) GRASP between *pdf*- and *ppk*-neurons. Scale bar: 15 μm. (**e**) Change in latency to the first night sleep episode in *4.1m* > shi^{ts} males and associated controls. n = 37-53. *p < 0.05, **p < 0.0005, ns – p > 0.05, Kruskal-Wallis test with Dunn's post-hoc test (**a**, **e**) or Mann-Whitney test (**c**).
